# Controle de Temperatura após Parada Cardíaca: Uma Revisão Narrativa na Perspectiva de um País em Desenvolvimento

**DOI:** 10.36660/abc.20240696

**Published:** 2025-08-14

**Authors:** Rafaela dos Santos Braga, Gisele Sampaio Silva, Pedro Kurtz, Sergio Timerman

**Affiliations:** 1 Universidade Federal de São Paulo São Paulo SP Brasil Universidade Federal de São Paulo, São Paulo, SP – Brasil; 2 Hospital Israelita Albert Einstein São Paulo SP Brasil Hospital Israelita Albert Einstein, São Paulo, SP – Brasil; 3 Instituto D’Or de Pesquisa e Ensino Rio de Janeiro RJ Brasil Instituto D’Or de Pesquisa e Ensino (IDOR), Rio de Janeiro, RJ – Brasil; 4 Hospital das Clínicas da Faculdade de Medicina da Universidade de São Paulo Instituto do Coração São Paulo SP Brasil Instituto do Coração do Hospital das Clínicas da Faculdade de Medicina da Universidade de São Paulo, São Paulo, SP – Brasil

**Keywords:** Síndrome Pós-parada Cardíaca, Controle de Temperatura Direcionado, Cuidados Pós-PCR

## Abstract

O gerenciamento direcionado de temperatura (GDT) é atualmente a única intervenção potencialmente neuroprotetora recomendada para cuidados pós-parada cardíaca. No entanto, há preocupações entre a comunidade científica em relação às evidências conflitantes que apoiam essa recomendação. Além disso, a maior parte dos ensaios incluídos em revisões sistemáticas que informam diretrizes e recomendações foram conduzidos em países desenvolvidos, com mix de casos e características dos pacientes que diferem significativamente da realidade de países em desenvolvimento como o Brasil. Temperaturas corporais elevadas induzem alterações na integridade da barreira hematoencefálica e aumentam a demanda cerebral por oxigênio. Podem causar desequilíbrios no metabolismo de oxigênio cerebral e no fluxo sanguíneo, levando à inflamação e apoptose. O objetivo principal do GDT é controlar as vias secundárias de lesão, evitando altas temperaturas. O GDT, anteriormente denominado hipotermia terapêutica, foi usado pela primeira vez para tratar lesão cerebral pós-parada cardíaca na década de 1950. Desde então, temos realizado ensaios clínicos relevantes sobre o GDT, com resultados conflitantes, como os seguintes: GDT1, estudo HACA, GDT2, estudo HYPERION e algumas metanálises mantiveram o manejo da temperatura após uma parada cardíaca em discussão. Além de individualizar a temperatura-alvo ideal para cenários clínicos e perfis de pacientes específicos, outros aspectos da administração de GDT de alta qualidade são cruciais. O momento de obtenção da temperatura-alvo, a duração do resfriamento, as taxas de reaquecimento e as práticas de sedação foram avaliados em ensaios clínicos recentes. Em conclusão, é crucial determinar a abordagem de GDT mais eficaz para alcançar os melhores resultados neurológicos possíveis, minimizando os potenciais efeitos adversos.

**Figure:**
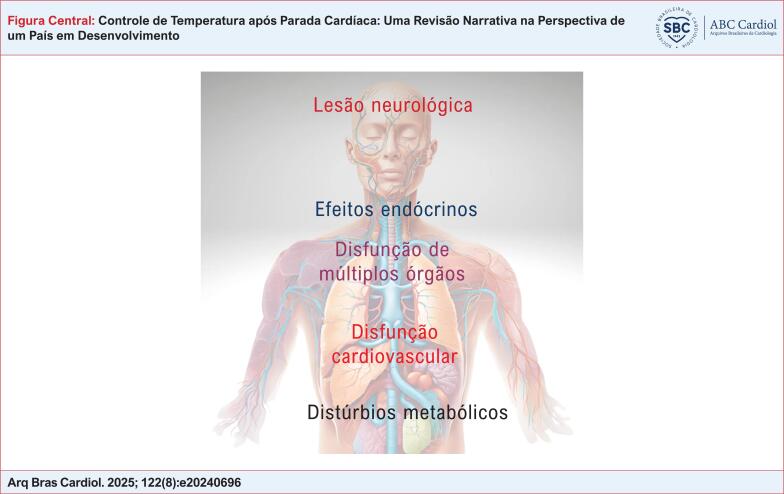


## Introdução

Reiniciar o coração após uma parada cardíaca pode resultar em uma condição conhecida como síndrome pós-parada cardíaca, que pode incluir lesão cerebral hipóxico-isquêmica (LCHI). Embora essa condição desencadeie uma resposta fisiopatológica complexa que pode levar à disfunção de múltiplos órgãos, a LCHI continua sendo a principal causa de morte naqueles que alcançam o retorno da circulação espontânea.^1^ Portanto, os cuidados pós-ressuscitação tornaram-se um foco importante e têm sido recomendados em diretrizes internacionais para o tratamento de parada cardíaca. O manejo pós-ressuscitação envolve várias terapias para otimizar a ventilação e a circulação, ao mesmo tempo em que previne danos neurológicos.^1^ O controle da temperatura é atualmente a única intervenção potencialmente neuroprotetora recomendada para os cuidados pós-parada cardíaca, mesmo com algumas controvérsias envolvendo o assunto. No entanto, há preocupações entre a comunidade científica em relação às evidências conflitantes que apoiam essa recomendação.^2 – 4^ Além disso, a maior parte dos ensaios incluídos em revisões sistemáticas que informam diretrizes e recomendações foi conduzida em países desenvolvidos, com uma combinação de casos e características dos pacientes que diferem significativamente da realidade de países em desenvolvimento, como o Brasil. Nesta revisão narrativa, discutiremos as controvérsias atuais em torno da eficácia do controle de temperatura quando aplicado para melhorar os resultados após uma parada cardíaca, com foco especial no papel desse tratamento no cenário clínico de um país de renda média com escassos dados disponíveis.

### Fisiopatologia

A parada cardíaca pode causar dois tipos de lesão neurológica. A primeira é o resultado da lesão hipoxêmica primária, enquanto temos uma segunda lesão que envolve desequilíbrios multissistêmicos após a restauração da circulação sistêmica.^3 – 5^ Acredita-se que a lesão primária causada pela parada cardíaca seja devida a despolarizações anóxicas, que interrompem o denominado gradiente iônico transmembrana. Isso resulta em despolarização descontrolada e disseminada, níveis variáveis de edema citotóxico e, na maioria dos casos, liberação de glutamato.^6 , 7^ A lesão cerebral secundária envolve vários fatores, como disfunção microcirculatória, produção de radicais livres de oxigênio, perda da autorregulação circulatória cerebral, excitotoxicidade, as cascatas de proteases podem ser ativadas e termina com edema cerebral. Insultos sistêmicos representados por hipotensão, hipoglicemia e hipertermia podem exacerbar ainda mais esses processos. Temperaturas corporais elevadas induzem alterações na integridade da barreira hematoencefálica e aumentam a demanda cerebral por oxigênio. Pode causar desequilíbrios no metabolismo do oxigênio cerebral e no fluxo sanguíneo, levando à inflamação e à apoptose.^6^ O objetivo principal do gerenciamento direcionado de temperatura (GDT) é controlar as vias secundárias de lesão, evitando altas temperaturas.^5^ A hipotermia parece atuar de forma diferente, controlando várias vias danosas simultaneamente para reduzir a morte celular no cérebro. Ela atenua as vias fisiopatológicas que levam à excitotoxicidade, apoptose, inflamação e produção de radicais livres, e afeta o fluxo sanguíneo, o metabolismo e a integridade da barreira hematoencefálica.^8 , 9^ A ilustração central mostra o sistema afetado pela síndrome pós-parada cardíaca.

### Cronograma e julgamentos importantes

O controle de temperatura, anteriormente conhecido como hipotermia terapêutica, foi utilizado pela primeira vez para tratar lesões cerebrais pós-parada cardíaca na década de 1950. Séries de casos clínicos da época sugeriram que pacientes submetidos à hipotermia entre 30 e 34°C por 24 a 72 horas apresentaram déficits neurológicos mínimos ou inexistentes após o reaquecimento.^7 , 10^ No entanto, os estudos apresentaram um viés de seleção, visto que nenhum controle recebeu controle de temperatura e todos os casos foram tratados no mesmo centro. Além disso, os efeitos nocivos do resfriamento sistêmico na época impediram o uso clínico generalizado desse tratamento.^11 , 12^

Após os resultados favoráveis do controle de temperatura em estudos com animais, vários estudos piloto não randomizados foram conduzidos. Em 2002, um ensaio clínico marcante foi publicado. O ensaio *Hypothermia After Cardiac Arrest* (HACA)^12^ incluiu pacientes de cinco países europeus com parada cardíaca (PCR) extra-hospitalar e ritmo cardíaco chocável (fibrilação ventricular e/ou taquicardia ventricular sem pulso). O ensaio randomizou 138 pacientes para não receber intervenção de temperatura, enquanto 137 pacientes receberam controle de temperatura de 32–34°C por 24 horas, seguido por 8 horas de reaquecimento passivo. Aos 6 meses, o grupo controle de temperatura apresentou melhores números de mortalidade (41% versus 55%) e melhores números de desfechos neurológicos favoráveis (Escala de Categorias de Desempenho Cerebral de Glasgow-Pittsburgh — CPC 1–2) (55% versus 39%) quando comparado ao grupo controle.^12^

Em 2013, o estudo GDT^13^ investigou se os benefícios do controle da temperatura poderiam ser alcançados com hipotermia mais branda. O GDT foi um estudo randomizado com 950 pacientes adultos após PCR e ritmo sem perfusão (exceto para assistolia em paradas não testemunhadas) que receberam controle de temperatura para 33 °C ou 36 °C por 24 horas. Foi seguido por reaquecimento lento. Além disso, houve uma prevenção ativa da febre até 72 horas após a parada. Não houve diferenças significativas na mortalidade ou desfecho neurológico ruim (CPC 3 a 5 ou escala de Rankin modificada [mRS] 4 a 6) entre os dois grupos em 6 meses. Portanto, o primeiro estudo GDT indicou que, em casos de PCR com causa cardíaca ou presumivelmente cardíaca, não houve benefício em manter uma temperatura de 33°C quando comparado a 36°C, desde que cuidados pós-parada cardíaca, reaquecimento lento controlado e neuroprognosticação fossem fornecidos. Como resultado dessas descobertas, muitos centros mudaram suas temperaturas-alvo para normotermia em vez de hipotermia.

Em 2017, Kirkegaard et al.^14^ incluíram 355 adultos em outro ensaio clínico randomizado com parada cardíaca extra-hospitalar e não encontraram diferença significativa no desfecho neurológico favorável em 6 meses para aqueles tratados por 48 horas (69%) versus 24 horas (64%) (diferença de 5%). Este foi um ensaio clínico internacional, paralelo, pragmático, multicêntrico, randomizado, de superioridade clínica, iniciado pelo pesquisador, com avaliação cega de desfechos, em 10 unidades de terapia intensiva (UTIs) de 10 hospitais universitários em 6 países europeus.^14^

O estudo HYPERION, em 2019, foi o primeiro ensaio randomizado a incluir parada cardíaca intra-hospitalar (PCRIH). Apenas ritmos não chocáveis foram incluídos. Neste estudo, 584 adultos que permaneceram inconscientes após o retorno da circulação espontânea foram selecionados para 33°C ou 37°C (+ / − 0,5°C) por 24 horas. Um reaquecimento lento controlado em pelo menos 24 horas e normotermia por mais 48 horas. No grupo hipotérmico, 10,2% dos indivíduos apresentaram independência em 90 dias versus 5,7% no grupo normotermia (p < 0,04). Por outro lado, é importante mencionar que o desfecho secundário, mortalidade em 90 dias, não diferiu entre os grupos.^15^

Publicado em 2021, o ensaio GDT2 testou a hipótese de que a prevenção ativa da febre não era inferior ao resfriamento até 33°C nos sobreviventes de uma parada cardíaca não hospitalar (PCRNH). Foi o maior ensaio multicêntrico internacional em parada cardíaca até o momento, com 1.861 PCRNH adultos inconscientes randomizados após qualquer ritmo inicial sem perfusão (exceto para paradas não testemunhadas com assistolia), 33°C ou manejo precoce da febre (ou seja, ≥ 37,8°C). Após 96 horas, a Neuroprognosticação foi padronizada, e o médico que a realizou foi cegado quanto à alocação do tratamento. Os pacientes no grupo de 33°C receberam hipotermia por 28 horas, seguida de reaquecimento de 0,3°C por hora e temperatura controlada entre 36,5 e 37,7°C por 72 horas. O grupo normotermia teve como meta < 37,8°C, e o resfriamento ativo foi realizado por resfriamento superficial ou endovascular após o uso de antipiréticos, 46% dos pacientes precisaram de algum antipirético para manter a temperatura alvo. Aos 6 meses, não houve diferenças significativas na mortalidade ou no desfecho neurológico quando comparamos os dois grupos.^16^

Nos últimos anos, tem havido uma demanda por revisão das conclusões dos ensaios iniciais sobre controle de temperatura. Isso se deve, em parte, à atualização da metodologia e da revisão estatística dos ensaios desde sua publicação. Alguns dos ensaios mais antigos não forneceram informações sobre como a febre foi evitada. Em comparação, os ensaios mais recentes incluíram protocolos detalhados e relataram o número de pacientes resfriados ativamente para atingir a normotermia usando dispositivos. Além disso, duas décadas se passaram desde os ensaios iniciais, durante os quais houve múltiplas mudanças no manejo, como melhorias na terapia intensiva, prognósticos neurológicos mais protocolizados e maiores taxas de sobrevida ao longo do tempo.^17^

Em 2022, Wolfrum S et al. publicaram um ensaio clínico randomizado que foi encerrado prematuramente por inutilidade. Eles compararam 32-34°C com normotermia e mortalidade em 180 dias; a mortalidade hospitalar e os desfechos funcionais foram melhores no grupo normotermia. Foi um ensaio clínico randomizado multicêntrico que comparou o controle da temperatura hipotérmica (32-34°C) por 24 h com normotermia após PCRIH em 11 hospitais na Alemanha. O desfecho primário foi a mortalidade por todas as causas após 180 dias.^18^

O *Capital Chill* é um ensaio clínico canadense, randomizado, duplo-cego, de superioridade clínica, unicêntrico, com um total de 389 pacientes com parada cardíaca fora do hospital. Sobreviventes comatosos de parada cardíaca fora do hospital apresentam altas taxas de mortalidade e lesões neurológicas graves, e o estudo pretendia comprovar que temperaturas mais baixas podem ter algum benefício nesse grupo de pacientes. No entanto, concluiu-se que, em sobreviventes comatosos de parada cardíaca fora do hospital, uma temperatura alvo de 31°C não reduziu significativamente a taxa de mortalidade ou desfecho neurológico desfavorável em 180 dias, em comparação com uma temperatura alvo de 34°C.^19^

Uma metanálise de rede^20^ publicada em 2021 revisou um total de dez ensaios clínicos randomizados escritos sobre o uso de controle de temperatura em sobreviventes de uma parada cardíaca de qualquer ritmo ou etiologia inicial, comparando vários alvos de controle de temperatura. Essa abordagem comparou intervenções com base em sua eficácia, uma vez que as comparações diretas eram limitadas. Sobreviver com bom resultado funcional na alta (CPC 1–2, mRS 0–3 ou avaliação clínica cega demonstrando incapacidade leve, moderada ou nenhuma) ou o último ponto de tempo registrado até 6 meses foi o resultado primário. Foi identificada evidência fraca de melhora com hipotermia leve (35–36 °C; OR 1,44 [IC 95% 0,74–2,80]), moderada (33–34°C; OR 1,34 [IC 95% 0,92–1,94]) ou profunda (31–32°C; OR 1,30 [IC 95% 0,73–2,30]) quando comparada à normotermia. Quando comparadas, a hipotermia moderada e a profunda não apresentaram benefício adicional na sobrevida ou no resultado funcional. No entanto, arritmias foram mais comuns no grupo de hipotermia profunda em comparação ao outro grupo que recebeu hipotermia moderada (OR 2,47 [IC 95% 1,25–4,88]). Não houve diferenças significativas em complicações clínicas como sangramento ou infecções nos grupos.^20^ Este método empregado na metanálise de rede é limitado pelas suposições de consistência de dados entre os estudos, que são necessárias para uma análise válida. Nesse contexto, uma metanálise bayesiana foi conduzida e publicada em 2022, empregando métodos que incorporam conhecimento prévio na análise.^21^ Essa abordagem fornece estimativas dos efeitos do tratamento juntamente com intervalos confiáveis que refletem a incerteza das estimativas. Os resultados deste estudo bayesiano convergiram para a mesma conclusão da metanálise em rede e de uma revisão sistemática atualizada do *International Liaison Committee on Resuscitation* (ILCOR) 2: não há evidências sólidas que sustentem o uso de hipotermia em níveis de temperatura de 32 a 34°C em comparação com o controle ativo da febre quanto ao risco de desfechos adversos após parada cardíaca. Em apoio adicional a essas recomendações, no final de 2023, outra metanálise focada em pacientes com parada cardíaca e ritmo cardíaco não chocável, incluídos em dois ensaios clínicos randomizados (GDT2 e Hyperion), não apresentou efeitos benéficos da hipotermia a 33°C, contrastando com os resultados positivos, embora com um tamanho de efeito pequeno, do próprio estudo Hyperion.^22^

Em contraste com todas as revisões e diretrizes publicadas recentemente, outra revisão sistemática atualizada, esta supervisionada pela Biblioteca Cochrane^3^ e publicada em 2023, concluiu que "evidências de baixa certeza sugerem que métodos convencionais de resfriamento para induzir hipotermia terapêutica leve podem melhorar o desfecho neurológico após parada cardíaca, especificamente se comparados à ausência de controle de temperatura". A revisão incluiu mais estudos e recebeu críticas de que alguns dos ensaios incluídos tinham métodos questionáveis em relação à randomização e à alocação de tratamento. No entanto, o nível de incerteza nos resultados e o fato de a maioria dos estudos positivos sobre hipotermia datarem de mais de 15 anos atrás reiteram que ainda existem lacunas consideráveis de conhecimento sobre qual é a abordagem ideal de controle de temperatura para cada sobrevivente de parada cardíaca.^22^

Essas lacunas, destacadas em uma revisão do *Science Advisory* da *American Heart Association* , têm implicações diretas para a prática clínica em países com limitações de recursos e escassez de dados clínicos sobre sobreviventes de parada cardíaca. A maioria dos pacientes tratados em ensaios clínicos randomizados de controle de temperatura, especialmente no GDT2, apresentava ritmos chocáveis e/ou etiologia cardíaca presumida para sua parada cardíaca.^1 , 2^ Além disso, 80% dos pacientes receberam ressuscitação cardiopulmonar por pessoas presentes, e quase metade necessitou de controle ativo de temperatura para evitar febre no grupo controle. Embora dados epidemiológicos concretos não estejam disponíveis, uma coorte brasileira^23^ publicada de 2.300 sobreviventes de parada cardíaca admitidos em terapia intensiva mostrou uma realidade notavelmente diferente: pelo menos dois terços tiveram parada cardíaca hospitalar, e apenas 13% foram admitidos na UTI após uma intervenção coronária, sugerindo uma etiologia cardíaca em uma minoria de indivíduos. De forma alarmante, apenas 1% da coorte recebeu controle ativo de temperatura, apesar de 1 em cada 10 hospitais responder que um protocolo de controle de temperatura havia sido implementado.^23^ Somada à falta de treinamento em RCP para pessoas presentes, à disponibilidade limitada de dispositivos com controle de temperatura por feedback e à ausência de centros de tratamento de parada cardíaca com grande volume de pacientes, a realidade do manejo pós-ressuscitação no Brasil permanece muito diferente daquela testada em ensaios clínicos randomizados. A Tabela 1 mostra os principais estudos sobre controle de temperatura.

**Tabela 1:** Revisões sistemáticas e metanálises em controle de temperatura

	Revisão sistemática / Metanálises				
Autores	Granfeldt et al.^2^	Arrich et al.^3^	Taccone et al.^38^	Aneman et al.^21^	Fernando et al.^20^
Ano de publicação	2023	2023	2023	2022	2021
Número de estudos	6	12	2	7	10
Número de pacientes	1719	3956	912	3792	4218
Tipos de parada cardíaca e ritmos	PCRNH e PCRIH	Não mencionado	PCRNH com ritmo não chocável	PCRNH e PCRIH com todos os ritmos iniciais	PCRNH
Metodologia	Metanálise de ensaios clínicos randomizados e quase randomizados desde 2021	Metanálise de ensaios clínicos randomizados e quase randomizados	Ensaios clínicos randomizados	Revisão sistemática e metanálise bayesiana de ensaios clínicos randomizados e quase randomizados	Revisão sistemática e metanálise de rede
Principais comparações	32-34°C vs normotermia	32-34°C vs normotermia	Hipotermia (temperatura alvo 33 °C) ou normotermia (temperatura alvo 36,5 a 37,7°C)	Qualquer temperatura GDT vs Sem GDT	Hipotermia leve, moderada e profunda vs normotermia
Principais descobertas	Resultado neurológico favorável na hipotermia (razão de risco: 1,14 [IC 95%: 0,98, 1,34])	Os participantes do grupo de hipotermia terapêutica tiveram maior probabilidade de alcançar um resultado neurológico favorável (razão de risco (RR) 1,41, intervalo de confiança (IC) de 95% 1,12 a 1,76	No último dia de acompanhamento, 386 de 429 no grupo de hipotermia (90,0%) e 413 de 463 no grupo de normotermia (89,2%) tiveram um resultado funcional desfavorável (RR com hipotermia, 0,99; IC de 95%, 0,87-1,15; p = 0,97)	A probabilidade posterior de nenhum benefício (RR ≥ 1) pelo GDT 32-34 °C foi de 24% para morte e 12% para desfecho neurológico desfavorável.	Sobrevivência com bom resultado funcional: hipotermia profunda (razão de chances 1,30, IC 95% 0,73-2,30), hipotermia moderada (OR 1,34, IC 95% 0,92-1,94) e hipotermia leve (OR 1,44, IC 95% 0,74-2,80)
Limitações	PCRNH foi incluído em todos os ensaios. O PCRNH foi incluído em apenas 1 de 6 ensaios	O uso de métodos inadequados para equilibrar os participantes entre os grupos de resfriamento e não resfriamento	Foram excluídos PCRIH e ritmos chocáveis	Os efeitos variáveis dentro da faixa hipotérmica não foram explorados	Ausência de fontes importantes de heterogeneidade clínica entre os ensaios em relação às características dos pacientes

PCRIH: parada cardíaca intra-hospitalar; PCRNH: parada cardíaca não hospitalar; GDT: gerenciamento direcionado de temperatura.

### Melhores práticas em controle de temperatura

Além de individualizar a temperatura-alvo ideal para cenários clínicos e perfis de pacientes específicos, outros aspectos da aplicação de controle de temperatura de alta qualidade são cruciais. O tempo de obtenção da temperatura-alvo, a duração do resfriamento, as taxas de reaquecimento e as práticas de sedação foram avaliados em estudos recentes.

Um estudo pivotal randomizou 789 adultos para duas estratégias de controle de temperatura, focadas na prevenção da febre e com duração variável – 36 versus 72 horas – e não encontrou diferença significativa no desfecho primário, em relação à mortalidade ou incapacidade grave dentro de 90 dias após o evento. Ambos os protocolos foram iniciados a 36°C nas primeiras 24 horas, sugerindo que a extensão da duração do controle de temperatura para prevenção da febre pode não impactar as taxas de mortalidade ou incapacidade grave. No entanto, a questão da duração ideal do controle de temperatura permanece em aberto. Os ensaios iniciais de hipotermia estabeleceram durações de 12 a 24 horas, equilibrando o potencial terapêutico com os efeitos colaterais. Após os ensaios de controle de temperatura, as diretrizes mudaram para recomendar pelo menos 24 horas de resfriamento, embora isso não tenha sido sustentado por evidências comparativas diretas. Um ensaio comparando 24 a 48 horas de controle de temperatura foi identificado e não relatou diferenças nos desfechos dos pacientes.^24 , 25^

Outros estudos, como os ensaios RINSE^26^ e PRINCESS,^27^ investigaram o tempo até o início do controle de temperatura. No RINSE, 1.198 pacientes com PCRNH foram randomizados para comparar o resfriamento intra-parada com o tratamento padrão. A porcentagem de pacientes com ritmos chocáveis que alcançaram o retorno da circulação espontânea no braço de resfriamento pré-hospitalar foi menor do que no braço de controle. Embora o grupo de resfriamento tenha chegado ao hospital com temperaturas mais baixas, isso não se traduziu em melhores resultados neurológicos. O ensaio PRINCESS repetiu esses achados; apesar do alcance mais rápido das temperaturas-alvo, nenhum benefício significativo na sobrevida ou nos resultados neurológicos foi observado. Nos grandes ensaios de controle de temperatura, o tempo para atingir a temperatura-alvo foi uma limitação comum, potencialmente impactando os resultados. Embora dados pré-clínicos sugiram que o resfriamento mais rápido melhora os resultados, isso não foi demonstrado em ensaios clínicos recentes.^26 , 27^

Embora as evidências atuais não favoreçam uma duração de controle de temperatura em detrimento de outra, há uma tendência que sugere que uma indução mais rápida à temperatura alvo pode ser benéfica. Isso reforça a necessidade de uma abordagem padronizada de cuidado que enfatize o manejo rápido da temperatura, o controle meticuloso da temperatura durante a manutenção e o manejo cuidadoso dos tremores, juntamente com o reaquecimento gradual e a normotermia controlada após o controle da temperatura. Tal protocolo visa aproveitar todo o potencial do controle de temperatura, ao mesmo tempo em que mitiga os efeitos adversos.^28 , 29^

Vários locais podem ser usados para monitoramento contínuo da temperatura central durante o controle de temperatura, incluindo sondas vesicais, esofágicas e retais. De acordo com a diretriz GDT da *Neurocritical Care Society* (NCS),^30^ as sondas esofágicas e vesicais são as mais precisas para refletir as temperaturas dos cateteres de artéria pulmonar. Além disso, foi recomendado que sondas de temperatura esofágica sejam usadas durante o controle de temperatura. É importante mencionar que o uso de sondas esofágicas é limitado a pacientes intubados, e há um incentivo crescente para abandonar o monitoramento da temperatura da bexiga para prevenir infecções do trato urinário associadas ao cateter. As sondas retais são as menos precisas, e as sondas de artéria temporal não são recomendadas para medição de temperatura, pois são imprecisas e não úteis para monitoramento contínuo.

Uma limitação importante de alguns dos ensaios clínicos incluídos é a ausência de um protocolo padronizado de controle de temperatura intra-hospitalar para garantir que todos os pacientes incluídos tenham recebido controle de temperatura no hospital. O controle de temperatura pode ser alcançado por diversos métodos, incluindo intervenções simples, como infusão rápida de fluidos frios e aplicação de compressas de gelo, mantas refrescantes ou almofadas adesivas de gel com mecanismos de feedback, ou dispositivos endovasculares automatizados.^30^

Vários métodos e dispositivos técnicos diferentes foram usados para induzir a hipotermia como alvo, mas não há consenso sobre o método de resfriamento ideal. De Fazio et al. concluíram em 2019 que os dispositivos de resfriamento endovascular poderiam ser mais precisos do que os métodos de superfície em pacientes resfriados a 33 °C após parada cardíaca fora do hospital. No entanto, os resultados foram semelhantes ao comparar os métodos de resfriamento, o que sugere que não há diferenças clinicamente relevantes nesse cenário.^31^ Uma revisão sistemática e metanálise encontrou 12 estudos com um total de 1573 participantes comparando a segurança e a eficácia dos dispositivos de resfriamento. Parece que os dispositivos intravasculares tendem a ser mais seguros em relação à mortalidade e aos desfechos neurológicos, com maior chance de arritmias, mas sem diferença significativa entre os grupos.^32^ A Atualização das Diretrizes da *American Heart Association* de 2015 para Ressuscitação Cardiopulmonar e Cuidados Cardiovasculares de Emergência afirma que o controle de temperatura, a melhor prática em controle de temperatura, deve ser com um mecanismo de controle de feedback de temperatura contínuo. Os métodos fáceis de usar são baratos, mas podem resultar em alterações e variações imprevisíveis na temperatura corporal, e a ausência de um mecanismo de controle por feedback de temperatura pode tornar o método pouco confiável. Dispositivos de resfriamento endovascular modernos ou dispositivos de resfriamento de superfície com mantas circulantes de água fria ou almofadas de hidrogel tendem a atingir a temperatura alvo e manter rapidamente as faixas de temperatura terapêutica desejadas por um período mais longo, utilizando um mecanismo de controle por feedback de temperatura. Até o momento, nenhuma revisão sistemática ou metanálise compara a eficácia desses dois tipos de dispositivos de resfriamento, ambos equipados com um mecanismo de controle por feedback de temperatura.

### Recomendações e práticas atuais

Em 2022, o *European Resuscitation Council* ^25^ publicou uma diretriz atualizada sobre o controle da temperatura em pacientes em coma após parada cardíaca. As recomendações mais importantes são o monitoramento contínuo da temperatura central e a prevenção da febre (definida como > 37,7 °C) por pelo menos 72 horas. Se necessário, recomenda-se o uso de medicamentos antitérmicos ou de um dispositivo de resfriamento. O estudo GDT2 foi a principal referência para essa recomendação.

As diretrizes esclarecem a insuficiência de evidências para recomendar ou não o resfriamento ativo de pacientes a 32–36 °C (o mesmo que nas diretrizes anteriores) ou o uso de resfriamento precoce após o retorno da circulação espontânea. Em resumo, elas recomendaram contra o reaquecimento ativo de pacientes comatosos hipotérmicos após parada cardíaca e recomendaram contra o uso de infusões de grande volume de fluido frio para resfriar pacientes imediatamente após atingir o RCE. No momento, temos uma atualização focada no controle de temperatura em andamento pela *American Heart Association* . Ela considerará novas evidências emergentes disponíveis desde a última diretriz sobre este tópico, em 2020. Nesse ínterim, um grupo consultivo científico da AHA concluiu que, para pacientes com características semelhantes às incluídas no estudo GDT2 – PCRNH de causa cardíaca ou desconhecida, excluindo aqueles com assistolia não testemunhada – controlar a temperatura central <37,5 °C é uma abordagem razoável e baseada em evidências.^33^ Eles também concordaram que, para o grupo mais amplo de sobreviventes de parada cardíaca com PCRNH, ou PCRIH com etiologia médica não cardíaca, a abordagem ideal de controle de temperatura permanece incerta. Nesse grupo, a temperatura-alvo individualizada pode ser definida entre 33°C e 37,5°C, com ênfase na administração de controle de temperatura de alta qualidade e suporte de terapia intensiva.

Apesar da descrição fisiopatológica apontar o uso da hipotermia como uma boa opção no manejo, os ensaios clínicos randomizados mais importantes não conseguiram demonstrar benefícios.

Recomenda-se como boa prática que a febre seja evitada ou controlada após uma parada cardíaca. A análise post-hoc do estudo FINNRESUSCI, um estudo observacional que avaliou a incidência de febre e os fatores preditores de febre após uma parada cardíaca, concluiu que metade dos pacientes não tratados com GDT desenvolveu febre, sendo a febre mais comum em pacientes com ritmo cardíaco não chocável, e reforçou que a febre pode estar relacionada a desfechos desfavoráveis.^34^

O estudo INTREPID randomizou 2.024 pacientes com AVC, correlacionando febre e desfechos funcionais. O estudo foi interrompido após uma análise interina planejada demonstrar a futilidade do desfecho secundário principal, concluindo que a normotermia preventiva reduziu a febre, mas não melhorou os desfechos funcionais. Isso demonstra que o conceito pós-parada cardíaca pode ser expandido para outras condições neurocríticas.^35^

O senso comum na metodologia e na amostra escolhida dos ensaios clínicos mais importantes sugere que a hipotermia pode ter lugar em algumas populações selecionadas.^36^

### Ensaios clínicos em andamento ou futuros sobre controle de temperatura após parada cardíaca

Uma tecnologia que permite o gerenciamento seletivo da temperatura cerebral com um dispositivo portátil foi publicada recentemente e seu uso pode ser razoável.^31^

Há três estudos promissores sobre controle de temperatura e resultados pós-parada cardíaca na fase de recrutamento.

O estudo STEP-CARE^32^ incluirá três intervenções diferentes com foco em metas de sedação, metas de temperatura e metas de pressão arterial média. Espera-se que o controle da temperatura seja estudado por meio do controle da febre, com ou sem um dispositivo controlado por feedback. Os participantes serão acompanhados por 30 dias e 6 meses. O desfecho primário será a sobrevida em 6 meses.

Outro ensaio clínico randomizado de recrutamento é o SELECT.^31^ O objetivo deste estudo será estimar a viabilidade e a segurança do desmame precoce do tratamento na UTI em pacientes após parada cardíaca e um padrão EEG favorável precoce (< 12 h). O delineamento do estudo é um delineamento cruzado randomizado por cluster com dois braços de tratamento. O contraste da intervenção será a interrupção precoce da sedação e controle da temperatura, com subsequente desmame da ventilação mecânica, se apropriado (grupo intervenção) versus o tratamento padrão, incluindo sedação e controle da temperatura por pelo menos 24 a 48 horas (grupo controle).

O estudo ICECAP^37^ medirá a influência da duração da hipotermia na eficácia em pacientes com parada cardíaca. Trata-se de um ensaio clínico multicêntrico e randomizado cujo objetivo é responder à questão de que o aumento da duração da hipotermia induzida está associado a uma taxa crescente de melhores desfechos neurológicos. A ideia é identificar a duração ideal da hipotermia induzida para neuroproteção em sobreviventes comatosos de parada cardíaca.

Mais recentemente, em dezembro de 2024, Skirifvars et al. propuseram uma revisão interessante mostrando os prós e os contras da GDT a 33 °C após parada cardíaca. Temos dados laboratoriais robustos, nenhum ensaio clínico sugerindo danos e o benefício relacionado à gravidade da lesão são aspectos positivos a serem considerados na GDT a 33 °C. Além disso, desvantagens como a falha em demonstrar benefício em ensaios clínicos randomizados recentes, o potencial dano na instabilidade cardíaca e o benefício em estudos com animais podem não ser replicados em humanos.^38^

## Conclusão

Em conclusão, é crucial determinar a abordagem de controle de temperatura mais eficaz para alcançar os melhores resultados neurológicos possíveis, minimizando os potenciais efeitos adversos. Mais pesquisas ainda são necessárias para refinar nossas decisões clínicas sobre a temperatura, duração e método de resfriamento ideais, bem como os dispositivos ideais para monitoramento contínuo da temperatura em diversos cenários clínicos. No entanto, as evidências atuais ainda sugerem que a prevenção da febre provavelmente não é inferior à hipotermia para muitos pacientes. Identificar quais subgrupos podem se beneficiar de temperaturas mais baixas continua sendo o desafio para estudos futuros. Particularmente no Brasil, aplicar as melhores práticas de controle de temperatura em um país vasto e desigual continua sendo a maior dificuldade.

Disponibilidade de Dados

Os conteúdos subjacentes ao texto da pesquisa estão contidos no manuscrito.
