# Supplementary material for: Controle de Temperatura após Parada Cardíaca: Uma Revisão Narrativa na Perspectiva de um País em Desenvolvimento
Source: Arq Bras Cardiol. 2025 Aug 14;122(8):e20240696. [Article in Portuguese] doi: 10.36660/abc.20240696 (PMC12671585; doi:10.36660/abc.20240696)
Supplement: Translation

# Translation: Temperature Control after Cardiac Arrest: A Narrative Review from a Developing Country Perspective

**DOI:** 10.36660/abc.20240696i

**Authors:** Rafaela dos Santos Braga, Gisele Sampaio Silva, Pedro Kurtz, Sergio Timerman

**Affiliations:** 1 São Paulo SP Brazil Universidade Federal de São Paulo, São Paulo, SP – Brazil; 2 São Paulo SP Brazil Hospital Israelita Albert Einstein, São Paulo, SP – Brazil; 3 Rio de Janeiro RJ Brazil Instituto D’Or de Pesquisa e Ensino (IDOR), Rio de Janeiro, RJ – Brazil; 4 São Paulo SP Brazil Instituto do Coração do Hospital das Clínicas da Faculdade de Medicina da Universidade de São Paulo, São Paulo, SP – Brazil

**Keywords:** Post Cardiac Arrest Syndrome, Targeted Temperature Management, Post Cardiac Arrest Management

## Abstract

Targeted temperature management (TTM) is currently the only potentially neuroprotective intervention recommended for post-cardiac arrest care. However, there are concerns among the scientific community regarding conflicting evidence supporting this recommendation. Moreover, the bulk of trials included in systematic reviews that inform guidelines and recommendations have been conducted in developed countries, with case mix and patient characteristics that significantly differ from the reality of developing countries such as Brazil. Elevated body temperatures induce changes in the blood-brain barrier integrity and increase the brain's demand for oxygen. They can cause imbalances in cerebral oxygen metabolism and blood flow, leading to inflammation and apoptosis. The primary aim of temperature control (TTM) is to control the secondary injury pathways by avoiding high temperatures. TTM, previously named therapeutic hypothermia, was first used to treat post-cardiac arrest brain injury in the 1950s. After that, we have been having relevant trials regarding TTM, with conflicting results as follows: TTM1, HACA study, TTM2, HYPERION study, and some meta-analyses kept the temperature management after a cardiac arrest in the discussion. In addition to individualizing the optimal target temperature for specific clinical scenarios and patient profiles, other aspects of high-quality TTM delivery are critical. The timing of target temperature achievement, duration of cooling, rewarming rates, and sedation practices have been evaluated in recent trials. In conclusion, it is crucial to determine the most effective TTM approach to achieve the best possible neurological outcomes while minimizing potential adverse effects.

## Introduction

Restarting the heart after cardiac arrest can result in a condition known as post-cardiac arrest syndrome, which may include hypoxic-ischemic brain injury (HIBI). Although this condition triggers a complex pathophysiological response that can lead to multiple organ dysfunction, HIBI remains the primary cause of death in those who achieve return of spontaneous circulation.^1^Therefore, post-resuscitation care has become an important focus and has been recommended in international guidelines for cardiac arrest treatment. Post-resuscitation management involves various therapies to optimize ventilation and circulation while preventing neurological damage.^1^Temperature control is currently the only potentially neuroprotective intervention recommended for post-cardiac arrest care, even with some controversies involving the issue. However, there are concerns among the scientific community regarding conflicting evidence supporting this recommendation.^2 – 4^Moreover, the bulk of trials included in systematic reviews that inform guidelines and recommendations have been conducted in developed countries, with case mix and patients’ characteristics that significantly differ from the reality of developing countries such as Brazil. In this narrative review, we will discuss the ongoing controversies around the efficacy of temperature control when applied to improve outcomes after cardiac arrest, with a special focus on the role of this treatment in the clinical scenario of a middle-income country with scarce data available.

### Pathophysiology

Cardiac arrest can cause two types of neurological injury. The first one is the result of the primary hypoxemic injury, while we have a second injury that involves multisystem imbalances after restoring systemic circulation.^3 – 5^The primary injury caused by cardiac arrest is believed to be due to anoxic depolarizations, which disrupt the named transmembrane ionic gradient. This results in uncontrolled spreading depolarization, variable levels of cytotoxic edema, and, in most cases, glutamate release.^6 , 7^Secondary brain injury involves various factors such as microcirculatory dysfunction, the production of oxygen-free radicals, loss of cerebral circulatory autoregulation, excitotoxicity, the protease cascades may be activated, and it ends up with cerebral edema. Systemic insults represented by hypotension, hypoglycemia, and hyperthermia can further exacerbate these processes. Elevated body temperatures induce changes in the blood-brain barrier integrity and increase the brain's demand for oxygen. It can cause imbalances in cerebral oxygen metabolism and blood flow, leading to inflammation and apoptosis.^6^The primary aim of temperature control (TTM) is to control the secondary injury pathways by avoiding high temperatures.^5^Hypothermia seems to act differently by controlling several damaging pathways simultaneously to reduce cell death within the brain. It mitigates pathophysiological pathways leading to excitotoxicity, apoptosis, inflammation, and free radical production, and affects the blood flow, metabolism, and blood–brain barrier integrity.^8 , 9^The central illustration shows the system affected by post-cardiac arrest syndrome.

### Timeline and important trials

Temperature control, previously known as therapeutic hypothermia, was first used to treat post-cardiac arrest brain injury in the 1950s. Clinical case series from that time suggested that patients submitted to hypothermia between 30 and 34°C for 24 to 72 hours had minimal or no neurological deficits after being rewarmed.^7 , 10^However, the studies presented a selection bias since no controls received temperature control, and all cases were treated at the same center. Additionally, the harmful effects of systemic cooling at that time prevented the widespread clinical use of this treatment.^11 , 12^

Following the favorable outcomes of temperature control in animal studies, various non-randomized pilot studies were conducted. In 2002, a landmark trial was published. The Hypothermia After Cardiac Arrest (HACA)^12^trial included patients from five European countries with out-of-hospital cardiac arrest (OHCA) and a shockable rhythm (ventricular fibrillation and/or pulseless ventricular tachycardia). The trial randomized 138 patients to receive no temperature intervention, while 137 patients received temperature control at 32–34°C for 24 hours, followed by 8 hours of passive rewarming. At 6 months, the temperature control group had better mortality numbers (41% versus 55%) and better numbers for favorable neurological outcomes (Glasgow-Pittsburgh Cerebral Performance Categories Scale—CPC 1–2) (55% versus 39%) when compared to the control group.^12^

In 2013, the TTM^13^trial investigated whether the benefits of temperature control could be accomplished with milder hypothermia. The TTM was a randomized trial with 950 adult patients after OHCA and non-perfusing rhythm (except for asystole in unwitnessed arrests) that received temperature control to either 33 °C or 36 °C for 24 hours. It was followed by slow rewarming. Also, there was an active prevention of fever until 72 hours post-arrest. It was no significant differences in mortality or poor neurological outcome (CPC 3 to 5 or modified Rankin scale [mRS] 4 to 6) between the two groups in 6 months. Therefore, the first TTM trial indicated that, in cases of OHCA with a cardiac or presumed cardiac cause, there was no benefit of maintaining a temperature of 33 °C when compared to 36 °C as long as post-cardiac arrest care, controlled slow rewarming, and neuroprognostication were provided. As a result of these findings, many centers shifted their target temperatures towards normothermia rather than hypothermia.

In 2017, Kikergraad et al.^14^enrolled 355 adults in another randomized clinical trial with out-of-hospital cardiac arrest, and found no significant difference in favorable neurologic outcome at 6 months for those treated for 48 hours (69%) vs 24 hours (64%) (difference, 5%). This was an international, investigator-initiated, blinded-outcome-assessor, parallel, pragmatic, multicenter, randomized clinical superiority trial in 10 intensive care units (ICUs) at 10 university hospitals in 6 European countries.^14^

The HYPERION study, in 2019, was the first randomized trial to include in-hospital cardiac arrest (IHCA). Only non-shockable rhythms were included. In this study, 584 adults who remained unconscious following return of spontaneous circulation were selected to 33 °C or 37 °C (+ / − 0.5 °C) for 24 hours. A controlled-slow rewarming in at least 24 hours and normothermia for an additional 48 hours. In the hypothermic group, 10.2% of subjects showed independence at 90 days versus 5.7% in the normothermia group (p <0,04). On the other hand, it is important to mention that the secondary outcome, mortality at 90 days, did not differ between groups.^15^

Published in 2021, the TTM2 trial tested the hypothesis that active prevention of fever was non-inferior to cooling until 33 °C in the survivors of an OHCA. It was the largest international multicenter trial in cardiac arrest to date, with 1861 unconscious adult OHCA randomized after any initial non-perfusing rhythm (except for unwitnessed arrests with asystole), either 33 °C or early management of fever (i.e., ≥ 37.8 °C). After 96 hours, Neuroprognostication was standardized, and the physician who performed it was blinded to treatment allocation. Patients in the 33 °C group received hypothermia for 28 hours, followed by rewarming from 0.3 °C per hour, and controlled temperature between 36.5 to 37.7 °C for 72 hours. The normothermia group targeted < 37.8 °C, and the active cooling was performed by using surface or endovascular cooling after antipyretics were tried— 46% of patients needed some antipyretics to maintain the target temperature. At 6 months, there were no significant differences in mortality or neurological outcome when we compared the two groups.^16^

Over the last few years, there has been a demand to review the conclusions of the initial trials on temperature control. This is partly because the methodology and statistical review of the trials have been updated since their publication. Some of the older trials did not provide information on how fever was avoided. In comparison, newer trials included detailed protocols and reported the number of patients actively cooled to achieve normothermia using devices. Additionally, two decades have passed since the initial trials, during which there have been multiple changes in management, such as improved intensive care, more protocolized neurological prognostication, and higher survival rates over time.^17^

In 2022, Wolfrum S et al. published a randomized controlled trial that was prematurely finished because of futility. They compared 32- 34 °C to normothermia and mortality in 180 days; hospital mortality and functional outcomes were better in the normothermia group. It was a multicenter, randomized controlled trial comparing hypothermic temperature control (32-34°C) for 24 h with normothermia after IHCA in 11 hospitals in Germany. The primary endpoint was all-cause mortality after 180 days.^18^

The Capital Chill is a Canadian, single-center, double-masked, randomized, clinical superiority trial with a total of 389 patients with out-of-hospital cardiac arrest. Comatose survivors of out-of-hospital cardiac arrest experience high rates of death and severe neurologic injury, and the trial intended to prove that lower temperatures may have some benefit in this group of patients. However, it was concluded that in comatose survivors of out-of-hospital cardiac arrest, a target temperature of 31 °C did not significantly reduce the rate of death or poor neurologic outcome at 180 days compared with a target temperature of 34°C.^19^

A network meta-analysis^20^published in 2021 reviewed a total of ten randomized clinical trials written about the use of temperature control in survivors of a cardiac arrest of any initial rhythm or etiology, comparing various targets of temperature control. This approach compared interventions based on their effectiveness since direct comparisons were limited. Surviving with good functional outcome at discharge (CPC 1–2, mRS 0–3, or blind clinical evaluation demonstrating mild, moderate, or no disability) or the latest time point recorded up to 6 months was the primary outcome. It was identified weak evidence of improvement with mild (35–36 °C; OR 1.44 [95% CI 0.74–2.80]), moderate (33–34 °C; OR 1.34 [95% CI 0.92–1.94]), or deep (31–32 °C; OR 1.30 [95% CI 0.73–2.30]) hypothermia when compared to normothermia. When compared, moderate and deep hypothermia showed no additional benefit on survival or functional outcome. Nevertheless, arrhythmias were more common in the group of deep hypothermia compared to the other group receiving moderate hypothermia (OR 2.47 [95% CI 1.25–4.88]). No significant differences in clinical complications as bleeding or infections, in the groups.^20^This method employed in the network meta-analysis is limited by the assumptions of data consistency among studies, which are necessary for valid analysis. In this context, a Bayesian meta-analysis was conducted and published in 2022, employing methods that incorporate prior knowledge into the analysis.^21^This approach provides estimates of treatment effects along with credible intervals reflecting the uncertainty of the estimates. The findings of this Bayesian study converged on the same conclusion as the network meta-analysis and from an updated systematic review of the International Liaison Committee on Resuscitation (ILCOR):^2^there is no strong evidence to support the use of hypothermia at temperature levels as 32–34°C compared to active control of fever about the risk of poor outcomes following cardiac arrest. In further support of these recommendations, in the end of 2023, another metanalysis focused on cardiac arrest patients with non-shockable rhythm included in 2 randomized trials (TTM2 and Hyperion) yielded no beneficial effects of hypothermia at 33°C, contrasting the positive results, albeit with a small effect size, of the Hyperion study itself.^22^

In contrast to all recently published reviews and guidelines, another updated systematic review, this one overseen by the Cochrane Library^3^and published in 2023, concluded that "low-certainty evidence suggests that conventional cooling methods to induce mild therapeutic hypothermia may improve neurological outcome after cardiac arrest, specifically if compared with no temperature management". It included more studies and received criticism that some of the included trials had questionable methods regarding randomization and treatment allocation. Nevertheless, the level of uncertainty in the findings and the fact that most of the positive studies on hypothermia date from more than 15 years ago reiterate that considerable knowledge gaps remain as to which is the optimal temperature control approach for which survivor of a cardiac arrest.^22^

These gaps, highlighted in a review by the Science Advisory from the American Heart Association, have direct implications for clinical practice in countries with resource limitations and scarcity of clinical data on cardiac arrest survivors. Most patients treated in randomized trials of temperature control, especially in TTM2, had shockable rhythms and/or presumed cardiac etiology for their cardiac arrest.^1 , 2^In addition, 80% of patients received bystander cardiopulmonary resuscitation, and almost half required active temperature control to avoid fever in the control group. Although hard epidemiological data are unavailable, a published Brazilian^23^cohort of 2,300 cardiac arrest survivors admitted to intensive care showed a strikingly different reality: at least two-thirds had in-hospital cardiac arrest, and only 13% were admitted to the ICU after a coronary intervention, suggesting a cardiac etiology in a minority of individuals. Alarmingly, only 1% of the cohort received active temperature control despite 1 in every 10 hospitals responding that a temperature control protocol had been implemented.^23^Combined with the lack of bystander CPR training, limited availability of temperature feedback-controlled devices, and absence of high-volume cardiac arrest treatment centers, the reality of post-resuscitation management in Brazil remains very different than the ones tested in randomized trials. Table 1 shows the main studies in temperature control.

**Table 1:** Systematic reviews and meta-analyses in temperature control

	Systematic review / Meta-analyses				
Authors	Granfeldt et al.^2^	Arrich et al.^3^	Taccone et al.^38^	Aneman et al.^21^	Fernando et al.^20^
Year published	2023	2023	2023	2022	2021
Number of studies	6	12	2	7	10
Number of patients	1719	3956	912	3792	4218
Types of cardiac arrest and rhythms	OHCA and IHCA	Not mentioned	OHCA with non-shockable rhythm	OHCA and IHCA with all initial rhythms	OHCA
Methodology	Meta-analysis of Randomized and quasi-randomized trials since 2021	Meta-analysis of randomized and quasi-randomized trials	Randomized clinical trials	Systematic review and Bayesian meta-analysis of randomized and quasi-randomized trials	Systematic review and network meta-analysis
Main comparisons	32-34°C vs normothermia	32-34°C vs normothermia	Hypothermia (target temperature 33 °C) or normothermia (target temperature 36.5 to 37.7 °C)	Any TTM temperature vs No TTM	Mild, moderate, and deep hypothermia vs normothermia
Main findings	Favorable neurological outcome in hypothermia (risk ratio: 1.14 [95%CI: 0.98, 1.34])	Participants in the therapeutic hypothermia group were more likely to reach a favorable neurological outcome (risk ratio (RR) 1.41, 95% confidence interval (CI) 1.12 to 1.76	On the last day of follow-up, 386 of 429 in the hypothermia group (90.0%) and 413 of 463 in the normothermia group (89.2%) had an unfavorable functional outcome (RR with hypothermia, 0.99; 95% CI, 0.87-1.15; P = .97)	The posterior probability for no benefit (RR ≥ 1) by TTM 32-34 °C was 24% for death and 12% for unfavorable neurological outcome.	Survival with good functional outcome: deep hypothermia (odds ratio 1.30, 95% CI 0.73-2.30), moderate hypothermia (OR 1.34, 95% CI 0.92-1.94), and mild hypothermia (OR 1.44, 95% CI 0.74-2.80)
Limitations	OHCA was included in all trials. IHCA was included in only 1 of 6 trials	The use of inadequate methods to balance participants between the cooling and no-cooling groups	IHCA and shockable rhythms were excluded	Variable effects within the hypothermic range were not explored	Lack of important sources of clinical heterogeneity across trials in relation to patient characteristics

OHCA: out-of-hospital cardiac arrest; IHCA: in-hospital cardiac arrest; TTM: targeted temperature management.

### Best practices in temperature control

In addition to individualizing the optimal target temperature for specific clinical scenarios and patient profiles, other aspects of high-quality temperature control delivery are critical. The timing of target temperature achievement, duration of cooling, rewarming rates, and sedation practices have been evaluated in recent trials.

A pivotal study randomized 789 adults to two temperature control strategies, focused on fever prevention, and varying in duration – 36 versus 72 hours – and found no significant difference in the primary outcome, about mortality or severe disability within 90 days post-event. Both protocols were initiated at 36°C for the first 24 hours, suggesting that the extension of temperature control duration for fever prevention may not impact the rates of death or severe disability. However, the question of optimal temperature control duration remains open. Initial trials of hypothermia set durations at 12 to 24 hours, balancing therapeutic potential against side effects. Following the temperature control trials, guidelines shifted to recommend at least 24 hours of cooling, though this was not underpinned by direct comparative evidence. One trial comparing 24 to 48 hours of temperature control was identified, and it reported no differences in patient outcomes.^24 , 25^

Other studies – i.e., RINSE^26^and PRINCESS^27^trials – investigated the timing until the temperature control starts. In RINSE, 1198 OHCA patients were randomized to compare intra-arrest cooling with standard care. The percentage of patients with shockable rhythms achieving return of spontaneous circulation in the pre-hospital cooling arm was lower than in the control arm. Although the cooling group arrived at the hospital with lower temperatures, this did not translate to improved neurological outcomes. The PRINCESS trial echoed these findings; despite faster achievement of target temperatures, no significant benefit in survival or neurological outcomes was observed. In the large temperature control trials, the time to achieve the target temperature was a common limitation, potentially impacting results. While preclinical data suggest that faster cooling improves outcomes, this has not been demonstrated in recent clinical trials.^26 , 27^

While current evidence does not favor one temperature control duration over another, there is a trend suggesting that faster induction to target temperature could be beneficial. This underscores the need for a standardized care approach that emphasizes prompt temperature management, meticulous temperature control during maintenance, and careful shivering management, coupled with gradual rewarming and controlled normothermia post-temperature control. Such a protocol aims to harness the full potential of temperature control while mitigating adverse effects.^28 , 29^

Multiple sites can be used for continuous core temperature monitoring during temperature control, including bladder, esophageal, and rectal probes. According to the Neurocritical Care Society (NCS) TTM guideline^30^, esophageal and bladder probes are the most accurate in reflecting temperatures of pulmonary artery catheters. Also, it was recommended that esophageal temperature probes should be used during temperature control. It is important to mention that the use of esophageal probes is limited to intubated patients, and there is increasing incentive to move away from bladder temperature monitoring to prevent catheter-associated urinary tract infections. Rectal probes are the least accurate, and temporal artery probes are not recommended for temperature measurement as they are inaccurate and not useful for continuous monitoring.

An important limitation of some of the included trials is the lack of a standardized in-hospital temperature control protocol to ensure that all included patients received temperature control in the hospital. Temperature control can be achieved by several different methods, including simple interventions such as rapid infusion of cold fluids and application of ice packs, cooling blankets or gel-adhesive pads with feedback mechanisms, or automated endovascular devices.^30^

Several different methods and technical devices have been used to induce hypothermia as the target, but there is no consensus on the optimal cooling method. De Fazio et al concluded in 2019 that endovascular cooling devices could be more precise than surface methods in patients cooled at 33°C after out-of-hospital cardiac arrest. However, the outcomes were similar when comparing the cooling methods, which suggests no clinically relevant differences in this setting.^31^A Systematic review and meta-analysis found 12 studies with a total of 1573 participants comparing the safety and effectiveness of cooling devices. It looks like intravascular devices tend to be safer regarding mortality and neurological outcomes, with a higher chance of arrhythmias, but no significant difference between the groups.^32^The 2015 American Heart Association Guidelines Update for Cardiopulmonary Resuscitation and Emergency Cardiovascular Care states that temperature control, the best practice in temperature control, should be with a continuous temperature feedback control mechanism. The easy-to-use methods are inexpensive, but they can result in unpredictable changes and variations in body temperature, and the lack of a temperature feedback control mechanism may render the method unreliable. Up-to-date endovascular cooling devices or surface cooling devices with cold-water circulating blankets or hydrogel pads tend to achieve the target temperature and maintain targeted therapeutic temperature rapidly ranges for a longer duration using a temperature feedback control mechanism. By the moment, no systematic review or meta-analysis compares the efficacy of these two types of cooling devices, both equipped with a temperature feedback control mechanism.

### Recommendations and current practice

In 2022, the European Resuscitation Council^25^released an updated guideline regarding temperature control in patients in coma after cardiac arrest. The most important recommendations are continuous monitoring of core temperature and prevention of fever (defined as > 37.7 °C) for at least 72 hours. If necessary, the use of antipyretic medications or a cooling device is recommended. The TTM2 trial was the main reference for this recommendation.

The guidelines shed light on the insufficient evidence to recommend for or against actively cooling patients to 32–36°C (the same as in prior guidelines) or the use of early cooling after return of spontaneous circulation. To sum up, they recommended against the active rewarming of hypothermic comatose patients following cardiac arrest and recommended against using large-volume infusions of cold fluid to cool patients immediately after achieving ROSC. By the moment, we have a focused update on temperature control in process by the American Heart Association. It will consider new emerging evidence available since the last guideline on this topic, in 2020. In this interim, a science advisory group from the AHA concluded that for patients with similar characteristics as those included in the TTM2 trial – OHCA of cardiac or unknown cause, excluding those with unwitnessed asystole–controlling core temperature <37.5 °C is a reasonable and evidence-based approach.^33^They also agreed that for the broader group of cardiac arrest survivors with IHCA, or OHCA with a noncardiac, medical etiology, the optimal temperature control approach remains uncertain. In this group, individualized target temperature can be defined between 33 °C and 37.5°C, with emphasis on the administration of high-quality temperature control and intensive care support.

Despite the pathophysiological description pointing to the use of hypothermia as a good option in management, the most important randomized trials failed to show benefits.

It is recommended as good practice that fever may be avoided or controlled in post-cardiac arrest. The post-hoc analysis of the FINNRESUSCI study, an observational study that assessed the incidence of fever and factors predicting fever after cardiac arrest, concluded that half of the patients not treated with TTM developed fever, fever was more common in patients with non-shockable rhythm, and reinforced that fever may be related to unfavorable outcomes.^34^

The INTREPID study in 2024 randomized patients with stroke, correlating fever and functional outcomes. The study was interrupted after a planned interim analysis demonstrated the futility of the principal secondary end point, concluding that preventive normothermia reduced fever but did not improve functional outcomes. Showing that the post-cardiac arrest concept may be expanded to other neurocritical ill conditions.^35^

A common sense in methodology and sample chosen of the most important clinical trials suggests that hypothermia may have a place in some selected populations.^36^

### Ongoing or future clinical trials on temperature control after cardiac arrest

A technology that allows selective brain temperature management with a portable device was recently published, and its use may be reasonable.^31^

There are three promisors trials about temperature control and post-cardiac arrest outcome in the recruitment phase.

The STEP-CARE trial^32^will include three different interventions focusing on sedation targets, temperature targets, and mean arterial pressure targets. Temperature control is expected to be studied through fever management with or without a feedback-controlled device. Participants will be followed up at 30 days and 6 months. The primary outcome will be survival at 6 months.

Another recruiting randomized trial is the SELECT.^31^The objective of this study will be to estimate the feasibility and safety of early weaning from ICU treatment in patients after cardiac arrest and an early (< 12 h) favorable EEG pattern. The study design is a cluster-randomized crossover design with two treatment arms. The intervention contrast will be early cessation of sedation and temperature control, with subsequent weaning from mechanical ventilation if appropriate (intervention group) vs. standard care, including sedation and temperature control for at least 24-48 hours (control group).

The ICECAP study^37^will measure the influence of hypothermia duration on efficacy in cardiac arrest patients. It is a multicenter and randomized clinical trial with the goal of answer the question that increasing durations of induced hypothermia are associated with an increasing rate of better neurological outcomes. The idea is to identify the optimal duration of induced hypothermia for neuroprotection in comatose survivors of cardiac arrest.

Most recently, in December 2024, Skirifvars et al proposed such an interesting review showing pros and cons with TTM at 33°C after cardiac arrest. We have strong laboratory data, no clinical trials suggesting harm, and the benefit related to the injury severity as positive things to think about in TTM at 33°C. Furthermore, disadvantages as the fail to show benefit in recent RCT trials, potential harm in cardiac instability, and the benefit in animal studies may not be replicated in humans.^38^

## Conclusion

In conclusion, it is crucial to determine the most effective temperature control approach to achieve the best possible neurological outcomes while minimizing potential adverse effects. Further research is still needed to refine our clinical decisions on the optimal temperature, duration, and method of cooling, as well as the ideal devices for continuous temperature monitoring in various clinical scenarios. Nonetheless, current evidence still suggests that preventing fever is likely non-inferior to hypothermia for many patients. Identifying which subgroups may benefit from lower temperatures remains the challenge for future trials. Particularly in Brazil, applying the best practice of temperature control in a vast and unequal country remains the biggest difficulty.

Data Availability

The underlying content of the research text is contained within the manuscript.
